# Impact of Manipulation Under Anesthesia on Functional Outcomes Following Total Knee Arthroplasty: A Systematic Review of the Knee Society Score (KSS), Oxford Knee Score (OKS), and Western Ontario and McMaster Universities Osteoarthritis Index (WOMAC)

**DOI:** 10.7759/cureus.106845

**Published:** 2026-04-11

**Authors:** Salis Aizaz Rasool, Mashal Mumtaz, Muhammad Zain Ul Abidin, Abdalla Kandeel, Shashwat Shetty, Rana Ahmed, Soliu Hamed Opeyemi, Shenouda R Shehata Abdelmesih, Ayan Ali

**Affiliations:** 1 Acute Medical Unit, Royal College of Surgeons in Ireland, Dudley, GBR; 2 Internal Medicine, University College of Medicine & Dentistry, Lahore, PAK; 3 Internal Medicine, University of Lahore, Lahore, PAK; 4 Trauma and Orthopaedics, Luton and Dunstable University Hospital, Luton, GBR; 5 Trauma and Orthopaedics, Dar El Shefaa Hospital, Kuwait, EGY; 6 Orthopaedics Department, Hillingdon Hospitals NHS Foundation Trust, Uxbridge, GBR; 7 Emergency Department, Hillingdon Hospitals NHS Foundation Trust, Uxbridge, GBR; 8 Surgery, Olabisi Onabanjo University Teaching Hospital, Sagamu, NGA; 9 Orthopaedics and Traumatology, Royal Gwent Hospital, Gwent, GBR; 10 Orthopedic Surgery, Dow University of Health Sciences, Karachi, PAK; 11 Orthopedic Surgery, Civil Hospital Karachi, Karachi, PAK

**Keywords:** arthrofibrosis, functional outcomes, knee society score, manipulation under anaesthesia, oxford knee score, postoperative stiffness, total knee arthroplasty, womac

## Abstract

Postoperative stiffness remains a significant complication following total knee arthroplasty (TKA), affecting functional recovery and patient satisfaction. Manipulation under anesthesia (MUA) is commonly employed when conservative measures fail, yet the impact on patient-reported outcomes remains unclear. This systematic review evaluated functional outcomes following MUA, focusing on the Knee Society Score (KSS), Oxford Knee Score (OKS), and Western Ontario and McMaster Universities Osteoarthritis Index (WOMAC) scores. A comprehensive search of PubMed, Embase, Scopus, and the Cochrane Library was conducted up to February 1, 2026, in accordance with Preferred Reporting Items for Systematic Reviews and Meta-Analyses (PRISMA) 2020 guidelines. Six studies, including retrospective and prospective cohorts and a systematic review, were included. Early MUA (<8-12 weeks) consistently achieved a superior range of motion (ROM) gains (+25° to +34°) compared to delayed interventions. Functional outcomes showed modest short-term improvements with sustained long-term benefits, and complications were rare. While ROM gains were reliably achieved, patient-perceived functional recovery depended on pain control, rehabilitation adherence, and individual factors. Early and standardized MUA is effective for improving mobility and functional recovery after TKA. Future high-quality prospective studies are warranted to refine timing, standardize techniques, and clarify the relationship between mechanical improvements and patient-centered outcomes.

## Introduction and background

Total knee arthroplasty (TKA) is one of the most successful orthopaedic procedures, providing substantial pain relief and functional restoration for patients with end-stage osteoarthritis. Despite advances in implant design, perioperative optimisation, and enhanced rehabilitation protocols, postoperative stiffness remains a clinically significant complication. The reported incidence of stiffness following primary TKA ranges from 1% to 6%, depending on the definition used (commonly flexion <90° or flexion contracture >10°) [1]. Arthrofibrosis after TKA leads to impaired gait, difficulty with stair climbing and rising from a seated position, and reduced overall patient satisfaction. Manipulation under anesthesia (MUA) is the most commonly employed intervention for early postoperative stiffness when conservative management fails. Reported rates of MUA after primary TKA vary between 1% and 5% in large registry and cohort studies [2]. Timing of intervention appears critical, with early MUA (typically within 12 weeks postoperatively) associated with greater improvements in range of motion (ROM) compared to delayed manipulation. Although ROM gains of 20°-40° are consistently reported, the relationship between mechanical improvement and functional recovery remains less clearly defined [3].

Functional outcomes after MUA are commonly evaluated using validated patient-reported outcome measures (PROMs), including the Knee Society Score (KSS), Oxford Knee Score (OKS), and Western Ontario and McMaster Universities Osteoarthritis Index (WOMAC). The KSS includes both a clinician-reported knee score assessing pain, stability, and alignment and a functional score evaluating walking distance and stair climbing, allowing both objective and functional assessment [4]. The OKS is a 12-item patient-reported outcome measure designed to assess pain and physical function in patients undergoing knee arthroplasty [5]. The WOMAC evaluates pain, stiffness, and physical function and is widely used to assess outcomes in patients with osteoarthritis of the knee and hip [6]. These instruments provide complementary perspectives on recovery, yet studies report variable improvements following MUA, and the extent to which improvements exceed minimal clinically important differences (MCIDs) is inconsistently described.

Given the heterogeneity in reported outcomes, timing of intervention, and methodological quality of available studies, a comprehensive evaluation of functional outcomes following MUA after primary TKA is warranted. The primary aim of this systematic review is to evaluate the impact of MUA on validated functional outcome measures (KSS, OKS, and WOMAC) following post-TKA stiffness. The secondary aims are to assess the influence of timing (early versus delayed MUA), quantify the range of motion (ROM) improvement, and evaluate short- and long-term complication rates associated with the procedure.

## Review

Materials and methods

Search Strategy

A systematic literature search was performed across four major databases, including PubMed, Embase, Scopus, and the Cochrane Library, to identify studies reporting functional outcomes following MUA after TKA. The search adhered to PRISMA 2020 guidelines to ensure transparency and methodological rigor [7]. Both controlled vocabulary terms (e.g., MeSH in PubMed, Emtree in Embase) and free-text keywords were used, combining concepts for “total knee arthroplasty” or “TKA”, “manipulation under anesthesia” or “MUA”, “arthrofibrosis”, and validated functional outcome measures including the KSS, OKS, and WOMAC. No language restrictions were applied. Studies published from database inception through February 1, 2026, were considered. Duplicate records were removed prior to screening, and reference lists of relevant reviews and included studies were hand-searched to capture additional eligible articles. An example of the full PubMed search strategy is provided in Supplementary Material (or Appendix), including Boolean operators, truncations, and filters, ensuring that the search is fully reproducible and transparent. This approach facilitated a comprehensive and systematic identification of relevant literature to assess the impact of MUA on both the objective ROM and patient-reported functional outcomes (PROMs).

Eligibility Criteria

Studies were considered eligible if they reported functional outcomes following MUA in patients with post-primary TKA stiffness, defined by limited knee ROM or flexion contracture. The eligibility was framed according to the PICO model [8]: the population included adult patients who had undergone primary TKA; the intervention was MUA; the comparison could include patients receiving delayed MUA, no intervention, or standard rehabilitation alone; and the outcomes of interest were validated patient-reported outcome measures, specifically KSS, OKS, and WOMAC scores, as well as ROM improvement. Both prospective and retrospective cohort studies, as well as systematic reviews reporting pooled outcomes, were included. Case reports, animal studies, editorials, and conference abstracts were excluded to focus on higher-quality evidence. Studies were required to provide sufficient follow-up data to allow assessment of functional outcomes post-MUA, and both early and late MUA interventions were considered.

Study Selection

All records identified from the search were first imported into a reference manager, and duplicates were removed systematically. Screening of titles and abstracts was performed independently by two reviewers to identify potentially relevant studies, followed by full-text review to determine final eligibility. Discrepancies between reviewers were resolved through discussion and consensus, with arbitration by a third reviewer if needed. The study selection process adhered to PRISMA guidelines, and a flow diagram was created to summarize the number of records identified, screened, assessed for eligibility, and included in the final review. Data extraction was conducted using a standardized form, capturing study design, population characteristics, timing and technique of MUA, functional outcomes, and reported complications. This rigorous selection process ensured that all included studies provided meaningful data on functional recovery after MUA following TKA.

Data Extraction

Data extraction was conducted using a standardized form developed to capture all relevant information from the included studies. Key data items collected included study design, level of evidence, patient demographics, sample size, timing and technique of MUA, ROM gains, functional outcomes measured by validated instruments (KSS, OKS, and WOMAC), and reported complications. Both short- and long-term follow-up outcomes were recorded to allow a comprehensive assessment of functional recovery and durability of improvements. Two reviewers independently extracted data to minimize errors and ensure accuracy, with discrepancies resolved through discussion and consensus. When necessary, corresponding authors were contacted for clarification or additional information. The systematic and structured approach to data extraction ensured consistency across studies and facilitated meaningful comparisons despite heterogeneity in methodology and outcome reporting.

Risk-of-Bias Assessment

The risk of bias of included studies was assessed using validated tools appropriate for study design. For observational cohort studies, the Newcastle-Ottawa Scale (NOS) was employed, evaluating selection of participants, comparability of groups, and outcome assessment [9]. For systematic reviews, the AMSTAR-2 tool was applied to assess methodological quality, including search strategy, study selection, data synthesis, and reporting transparency [10]. Two independent reviewers conducted the assessments, with disagreements resolved through discussion or arbitration by a third reviewer. The risk-of-bias evaluation considered both internal validity and the potential for confounding, recognizing that most studies were non-randomized. The results of this assessment informed the interpretation of outcomes, highlighting areas where methodological limitations might influence the reliability of reported functional improvements following MUA.

Data Synthesis

Given the heterogeneity in study design, patient populations, MUA protocols, and reporting of outcomes, a narrative synthesis was conducted rather than a meta-analysis. Studies were grouped according to timing of intervention (early versus delayed MUA) and type of outcome reported, including objective ROM and validated functional scores, such as the KSS, OKS, and WOMAC. Trends in functional improvement and complications were summarized across studies, highlighting consistencies and discrepancies in results. The narrative approach allowed the identification of factors influencing outcomes, including patient demographics, rehabilitation protocols, and MUA technique. Short- and long-term outcomes were described, and the clinical significance of ROM gains and PROM changes relative to MCIDs was assessed. This approach provided a comprehensive understanding of the impact of MUA on both mechanical and functional recovery following TKA. The systematic review by Gu et al. (2018) was included to provide contextual synthesis and highlight overall trends in ROM and functional outcomes. To avoid duplication bias, quantitative data from this review were not combined with primary studies, and overlapping patient populations were identified through cross-referencing.

Registration 

This systematic review was not prospectively registered in PROSPERO, which represents a limitation in terms of formal protocol transparency. Nonetheless, the review was conducted strictly according to PRISMA 2020 guidelines, with a predefined protocol outlining the search strategy, eligibility criteria, study selection, data extraction, and risk-of-bias assessment. All methodological steps were planned a priori and applied consistently to minimize bias, ensuring a systematic and reproducible approach despite the absence of formal registration. This limitation is acknowledged to maintain transparency regarding potential influences on post hoc decisions during the review process.

Results

Study Selection Process

**Figure 1:**
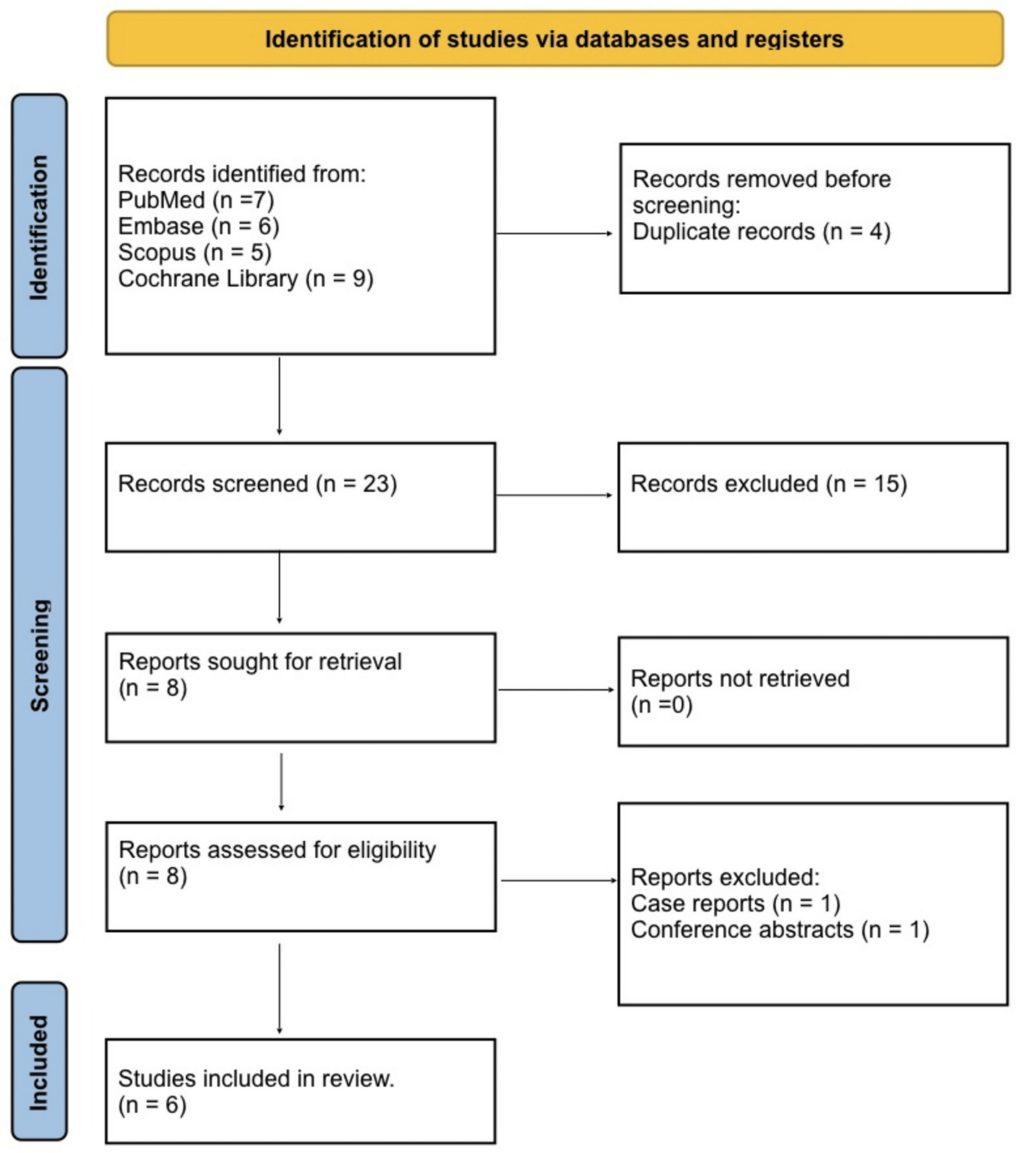
Preferred Reporting Items for Systematic Reviews and Meta-Analyses (PRISMA) 2020 flow diagram

Figure 1 shows that a total of 27 records were initially identified from the comprehensive search across multiple databases, including PubMed, Embase, Scopus, and the Cochrane Library, with seven records from PubMed, six from Embase, five from Scopus, and nine from the Cochrane Library. Before screening, duplicate records were removed, resulting in four duplicates being excluded. The remaining 23 records were screened by title and abstract for relevance, of which 15 were excluded due to not meeting the inclusion criteria. Eight full-text reports were sought for retrieval, all of which were successfully obtained and assessed for eligibility. During full-text review, one report was excluded because they were case reports, and one was a conference abstract, leaving a total of six studies that met all eligibility criteria and were included in the final systematic review. This process ensured a rigorous and transparent selection of studies in accordance with PRISMA 2020 guidelines.

Characteristics of the Selected Studies

Table 1 summarizes six studies evaluating outcomes following MUA for post-primary TKA stiffness. Early MUA (<8-12 weeks) consistently resulted in superior ROM gains, ranging from +25° to +34° [11], whereas delayed MUA achieved smaller improvements (+22° to +25°). ROM gains were generally maintained at long-term follow-up, demonstrating the durability of early intervention [11]. PROMs, including the KSS, OKS, and WOMAC, showed more modest short-term improvements. For example, Newman et al. reported comparable KSS and WOMAC outcomes between MUA and matched controls [12], while Sala J et al. [13] and Rantasalo et al. [14] observed minimal short-term PROM changes despite consistent ROM improvements (~26°), with some studies demonstrating more favorable long-term functional outcomes [14]. Gu et al., in a systematic review pooling 22 studies, reported mean ROM gains of 25-35° and general improvements in PROMs, with low complication rates [15]. Across all studies, MUA was performed under general or spinal anaesthesia using standardized flexion-extension protocols, and adverse events were rare, highlighting early MUA as effective for restoring mobility, while functional recovery may be influenced by patient-specific factors, rehabilitation adherence, and pain control [11,16]. Findings from Gu et al. (2018) corroborate trends observed in the primary studies, but data from this review were not combined with the included cohorts to prevent duplication of outcomes. The included studies reported improvements in the KSS, OKS, and WOMAC scores; however, MCIDs were not consistently reported, and explicit MCID-based analysis could not be performed.

**Table 1:** Characteristic of the included studies MUA: manipulation under anesthesia, TKR: total knee replacement, ROM: range of motion, KSS: Knee Society Score, OKS: Oxford Knee Score, WOMAC: Western Ontario and McMaster Universities Osteoarthritis Index, PROM: patient-reported outcome measure, GA: general anesthesia

Study	Study design	LOE	Population	n (MUA / control)	Timing of MUA	MUA technique	ROM gain	PROMs (KSS, OKS, and WOMAC)	Short-term complications	Long-term outcomes	Key clinical interpretation
Issa et al., 2014 [11]	Retrospective cohort	III	Post-TKR arthrofibrosis (<90° flexion)	144 / 0	Early <12 wks vs Late >12 wks	GA; gradual controlled flexion; patellar mobilisation; no arthroscopy	+34° (early) vs +25° (late)	KSS ↑ Knee +20 / Function +18	Haemarthrosis <2%, pain flare	ROM maintained at 2 yrs	Early MUA reliably improves ROM; functional recovery also improved
Newman et al., 2018 [12]	Matched cohort	III	Post-primary TKR stiffness	38 / 38	Mean 10 wks	GA or spinal; progressive flexion technique	+27°	KSS/WOMAC comparable to matched controls	None	No increased revision risk	Functional parity achievable; ROM reliably improved
Sala J et al., 2022 [13]	Retrospective cohort	III	Post-primary TKR stiffness	145 / 0	Mean ~13 wks	GA or spinal; standardised manipulation protocol	+26° (95% CI 23–29°)	KSS/OKS minimal short-term change; early MUA associated with greater flexion gain	None	ROM maintained at follow-up	MUA reliably improves ROM; PROM improvements modest in short-term
Rantasalo et al., 2022 [14]	Retrospective cohort	III	Post-TKR stiffness	145 / 0	Mean 13 wks	GA/spinal; standardised manipulation	+26°	KSS/OKS minimal short-term change; PROM improved long-term	None	ROM maintained	Early MUA improves ROM; long-term PROMs improved
Gu et al., 2018 [15]	Systematic review	III–IV	Post-TKR stiffness	22 studies pooled	Variable	GA or spinal; protocols varied	+25–35° (mean)	KSS, OKS, WOMAC improved post-MUA	Rare fractures, haemarthrosis	ROM and functional scores maintained 1–5 yrs	Confirms early MUA improves ROM; PROM improvements observed with low complications
Rahardja et al., 2023 [16]	Prospective cohort	III	Early post-TKR stiffness	131 / 0	Early <8 wks vs Late	GA or spinal; standardised flexion-extension cycles	+28° early vs +22° late	KSS improved; PROM improvements significant	Minor pain flare	ROM maintained at 1 yr	Early MUA provides superior ROM and functional recovery

Risk-of-Bias Assessment

Table 2 shows that studies were generally moderate, reflecting the predominance of observational designs. Issa et al. (2014) [11] and Sala et al. (2022) [13] were retrospective cohorts with moderate selection bias, objective measurement of ROM, and validated patient-reported outcome measures (PROMs), although the absence of randomisation and control groups limited comparative inference. Newman et al. (2018) [12] used a matched cohort design, reducing selection bias and enhancing comparability, yet a blinded assessment was not stated. Rantasalo et al. (2022) [14] had limited follow-up (two months), leading to a moderate-high overall risk, while Rahardja et al. (2023) [16] performed multivariate adjustment to strengthen validity despite residual confounding. Gu et al. (2018) [15], in a systematic review, assessed using AMSTAR-2, showed a low risk in search and selection but a moderate overall risk due to heterogeneity and lack of meta-analysis, as shown in Table 3. These assessments indicate that while outcomes are generally reliable, limitations inherent to non-randomised designs and short-term follow-up should be considered when interpreting functional improvements. Most included studies were retrospective observational cohorts, with several lacking control groups and having variable follow-up durations. These characteristics introduce potential confounding, selection bias, and heterogeneity in outcome reporting, limiting the reliability and generalizability of the findings.

**Table 2:** Risk-of-bias assesment of the included studies by the NOS assesment tool The Newcastle-Ottawa Scale (NOS) evaluates three domains: selection (maximum 4 stars), comparability (maximum 2 stars), and outcome (maximum 3 stars). Higher scores indicate lower risk of bias.

Study	Study design	Risk of bias tool	Selection (max 4★)	Comparability (max 2★)	Outcome (max 3★)	Total (max 9★)	Overall confidence (AMSTAR-2)*	Justification
Issa et al., 2014 [11]	Retrospective cohort	Newcastle–Ottawa Scale (NOS)	★★★★	★	★★★	8★	NA	Clearly defined cohort with objective ROM measurement and validated KSS; non-randomised timing groups limit comparability.
Newman et al., 2018 [12]	Matched cohort	Newcastle–Ottawa Scale (NOS)	★★★	★★	★★★	8★	NA	Matched control design improves comparability; validated KSS and WOMAC used; retrospective nature without randomisation.
Sala et al., 2022 [13]	Retrospective cohort	Newcastle–Ottawa Scale (NOS)	★★★	★	★★★	7★	NA	Large sample size and validated PROMs; absence of control group reduces comparability.
Rantasalo et al., 2022 [14]	Retrospective cohort	Newcastle–Ottawa Scale (NOS)	★★★	★	★★	6★	NA	Validated PROMs used; short follow-up duration limits outcome domain scoring.
Rahardja et al., 2023 [16]	Prospective cohort	Newcastle–Ottawa Scale (NOS)	★★★★	★★	★★★	9★	NA	Prospective design with multivariable adjustment enhances comparability; adequate follow-up and objective outcome assessment.

**Table 3:** Risk-of-bias assessment of the included studies by AMSTAR-2

Study	Study design	Risk of bias tool	Overall confidence	Key considerations
Gu et al., 2018 [15]	Systematic Review	AMSTAR-2	Moderate	Comprehensive search strategy and structured study selection; however, heterogeneity among included studies and absence of meta-analysis limited methodological confidence.

Discussion

This systematic review demonstrates that MUA is an effective intervention for managing postoperative stiffness following TKA. Across the included studies, early MUA, performed within eight to 12 weeks postoperatively, consistently resulted in superior improvements in knee ROM, with gains ranging from +25° to +34° [11,16]. Delayed MUA generally achieved smaller ROM improvements (+22° to +25°). These mechanical gains were maintained over long-term follow-up, up to two to five years, highlighting the durability and reliability of early intervention. Consistent improvement in ROM across both retrospective and prospective cohorts, as well as pooled evidence from systematic reviews [15], underscores early MUA as a critical component in restoring functional knee mechanics and mobility efficiently.

While the objective improvements in ROM were substantial, PROMs, including the KSS, OKS, and WOMAC, demonstrated more modest and variable gains. Several studies, including Sala J et al. [13] and Rantasalo et al. [14], reported minimal short-term improvement in PROMs despite significant mechanical gains, suggesting that enhanced knee flexion alone may not fully translate into perceived functional benefit. Factors contributing to this discrepancy likely include persistent postoperative pain, quadriceps weakness, incomplete adherence to physiotherapy, and psychosocial elements such as motivation, expectations, and coping strategies. Nonetheless, longer-term follow-up in some cohorts demonstrated meaningful improvements in PROMs, particularly when MUA was performed early and paired with structured rehabilitation programs. Matched cohort studies, such as Newman et al. [12], indicate that functional parity with non-stiff TKA patients is achievable, reinforcing that early and proactive intervention can optimize both objective and subjective recovery. While improvements in functional scores were observed, the lack of consistent MCID reporting limits conclusions regarding the clinical significance of these changes.

The safety profile of MUA was favorable across studies. Short-term adverse events were uncommon and generally minor, including transient pain flares and occasional haemarthrosis, with no significant long-term sequelae or increased risk of revision procedures [14,15]. This supports the view that MUA is a low-risk intervention when performed by trained clinicians following standardized protocols, further strengthening its role as a first-line treatment for postoperative stiffness after TKA. Despite these encouraging findings, limitations in the current literature warrant caution. Most included studies were retrospective in nature, with moderate risk of bias, small sample sizes, and variable follow-up durations. Heterogeneity in MUA techniques, timing definitions, and outcome assessment methods, including inconsistent reporting of MCIDs for PROMs, reduces comparability and limits the ability to draw definitive conclusions. Furthermore, the majority of studies did not account for confounding factors such as comorbidities, preoperative ROM, or intensity of physiotherapy, which may influence both mechanical and functional recovery. While improvements in ROM and functional scores were observed, the predominance of retrospective study designs, lack of controls, and heterogeneity in outcomes limit the strength of evidence. Conclusions regarding clinical effectiveness should therefore be interpreted with caution.

Future research should prioritize high-quality, prospective, multicenter randomized trials to determine the optimal timing for MUA, standardize procedural protocols, and identify predictors of both ROM and functional recovery. Integrating objective measures of knee mechanics with validated patient-centered outcomes and quality-of-life assessments will provide a more comprehensive understanding of MUA’s benefits. In addition, exploring strategies to enhance PROM improvements, such as multimodal pain management, targeted physiotherapy, and patient education, may help align mechanical gains with perceived functional recovery, ultimately improving patient satisfaction and long-term outcomes following TKA.

## Conclusions

This review suggests that MUA may be associated with improved ROM in patients experiencing postoperative stiffness following TKA, particularly when performed early within eight to 12 weeks. Observed improvements in PROMs, including KSS, OKS, and WOMAC scores, were generally modest and variable, and may be influenced by factors such as pain management, rehabilitation adherence, and individual patient characteristics. Reported complications were uncommon, and ROM gains appeared to be maintained in the limited long-term follow-up available. Given that the evidence consists predominantly of small observational studies with heterogeneous protocols and outcomes, these findings should be interpreted as suggestive rather than definitive. Future high-quality, prospective, randomized studies are needed to better define the optimal timing, standardize procedural techniques, and clarify the relationship between mechanical improvements and patient-centered functional outcomes, to more reliably guide clinical decision-making in post-TKA stiffness.

## Appendices

**Table 4:** PubMed search strategy

Component	Search terms
Population	"Arthroplasty, Replacement, Knee"[MeSH] OR "total knee arthroplasty" OR "total knee replacement" OR TKA OR TKR
Intervention	"Manipulation, Orthopedic"[MeSH] OR "manipulation under anesthesia" OR MUA
Condition	"Arthrofibrosis"[MeSH] OR arthrofibrosis OR stiffness OR "joint stiffness"
Outcomes	"Knee Society Score" OR KSS OR "Oxford Knee Score" OR OKS OR WOMAC OR "Western Ontario and McMaster Universities Osteoarthritis Index"
Final Boolean strategy	(Population) AND (Intervention) AND (Condition) AND (Outcomes)
Filters applied	Humans; No language restrictions; From database inception to February 1, 2026

## References

[1] Archunan M, Swamy G, Ramasamy A (2021). Stiffness after total knee arthroplasty: prevalence and treatment outcome. Cureus.

[2] Baum KS, Luo TD, Comadoll S, Marois A, Langfitt M, Shields J (2018). Alternative technique for knee manipulation under anesthesia. Arthroplast Today.

[3] Namba RS, Inacio M (2007). Early and late manipulation improve flexion after total knee arthroplasty. J Arthroplasty.

[4] (2012). The 2011 Knee Society Knee Scoring System©. https://share.google/HnN8QQjxPjgNsecKO.

[5] The Oxford Knee Score (OKS). https://innovation.ox.ac.uk/outcome-measures/oxford-knee-score-oks/.

[6] Stucki G, Meier D, Stucki S, Michel BA, Tyndall AG, Dick W, Theiler R (1996). Evaluation of a German version of WOMAC (Western Ontario and McMaster Universities) Arthrosis Index [Article in German]. Z Rheumatol.

[7] Page MJ, McKenzie JE, Bossuyt PM (2021). The PRISMA 2020 statement: an updated guideline for reporting systematic reviews. Syst Rev.

[8] Brown D (2020). A review of the PubMed PICO tool: using evidence-based practice in health education. Health Promot Pract.

[9] Stang A (2010). Critical evaluation of the Newcastle-Ottawa scale for the assessment of the quality of nonrandomized studies in meta-analyses. Eur J Epidemiol.

[10] Shea BJ, Reeves BC, Wells G (2017). AMSTAR 2: a critical appraisal tool for systematic reviews that include randomised or non-randomised studies of healthcare interventions, or both. BMJ.

[11] Issa K, Banerjee S, Kester MA, Khanuja HS, Delanois RE, Mont MA (2014). The effect of timing of manipulation under anesthesia to improve range of motion and functional outcomes following total knee arthroplasty. J Bone Joint Surg Am.

[12] Newman ET, Herschmiller TA, Attarian DE, Vail TP, Bolognesi MP, Wellman SS (2018). Risk factors, outcomes, and timing of manipulation under anesthesia after total knee arthroplasty. J Arthroplasty.

[13] Sala J, Jaroma A, Sund R, Huopio J, Kröger H, Sirola J (2022). Manipulation under anesthesia after total knee arthroplasty: a retrospective study of 145 patients. Acta Orthop.

[14] Rantasalo MT, Palanne RA, Saini S, Vakkuri AP, Madanat R, Noora SK (2022). Postoperative pain as a risk factor for stiff knee following total knee arthroplasty and excellent patientreported outcomes after manipulation under anesthesia. Acta Orthop.

[15] Gu A, Michalak AJ, Cohen JS, Almeida ND, McLawhorn AS, Sculco PK (2018). Efficacy of manipulation under anesthesia for stiffness following total knee arthroplasty: a systematic review. J Arthroplasty.

[16] Rahardja R, Mehmood A, Coleman B, Munro JT, Young SW (2023). Early manipulation under anaesthesia for stiffness following total knee arthroplasty is associated with a greater gain in knee flexion. Knee Surg Sports Traumatol Arthrosc.

